# Real‐world epidemiology and treatment patterns of patients with locally advanced or metastatic urothelial carcinoma: Retrospective analysis of Diagnosis Procedure Combination claims data in Japan

**DOI:** 10.1111/iju.15450

**Published:** 2024-03-12

**Authors:** Keiko Asakawa, Miina Waratani, Olivia Massey, Tim Holbrook, Makoto Kondo, Atsushi Saito, Hiroyuki Nishiyama

**Affiliations:** ^1^ Department of Global Medical Affairs Japan Astellas Pharma, Inc. Tokyo Japan; ^2^ Secondary Data Evidence Generation, Adelphi Real World Bollington UK; ^3^ Department of Urology Tsukuba University Tsukuba Japan

**Keywords:** epidemiology, Japan, locally advanced/ metastatic urothelial carcinoma, real‐world, retrospective analysis, treatment patterns

## Abstract

**Objectives:**

Evaluate real‐world epidemiologic trends and treatment patterns in newly diagnosed patients with locally advanced or metastatic urothelial carcinoma (la/mUC) in Japan.

**Methods:**

This retrospective analysis included adults with newly diagnosed la/mUC in Japan (January 2015–December 2019) from a nationwide‐linked electronic medical record Diagnostic Procedure Combination claims dataset. Outcomes included epidemiologic trends (incidence and prevalence), baseline demographics, clinical characteristics, and treatment patterns in newly diagnosed patients with la/mUC before (2015–2017) and after (2018–2019) approval of pembrolizumab in Japan.

**Results:**

Of 975 patients included, 76.4% were men; 71.6% were aged 70 years or older. Most cases (70.5%) were of the bladder. Between 2015 and 2019, the annual age‐adjusted incidence increased from 6.8 to 12.4 per 100 000; the annual age‐adjusted period prevalence increased from 13.0 to 25.2 per 100 000; and 307 (31.5%) and 668 (68.5%) patients were diagnosed from 2015 to 2017 and 2018 to 2019, respectively. Overall, 731 (75%) patients received systemic anticancer therapy; all received 1 line and 50.2% received 2 lines of therapy; 78.3% of patients received gemcitabine plus platinum‐based therapy and 2.2% received pembrolizumab as first‐line treatment. First‐line treatment rates increased from 69.4% to 77.5% after pembrolizumab approval. Of 367 patients who received second‐line treatment, 22.3% received gemcitabine plus platinum‐based therapy; 14.7% received pembrolizumab.

**Conclusions:**

In the Japanese regions considered, incidence and prevalence of newly diagnosed la/mUC increased over time and first‐line treatment with pembrolizumab increased after approval.

**Abbreviations & Acronyms**
ADC: antibody drug conjugate
ECOG PS: Eastern Cooperative Oncology Group performance status
ICD‐10: *International Classification of Diseases, Tenth Revision*
la/mUC: locally advanced or metastatic urothelial carcinoma
MEDIS‐DC: Medical Information System Development Center
PD‐1/L1: programmed cell death protein 1/ligand 1
UC: urothelial carcinoma

## INTRODUCTION

Worldwide, an estimated 212 536 deaths related to bladder cancer occurred in 2020.^1^ In Japan, the estimated numbers of new cases of bladder cancer in 2020 were 29 010 for men and 7947 for women, with age‐standardized incidence rates of 14.4 and 2.9 per 100 000 persons, representing the sixth and thirteenth most common cancer in men and women, respectively.^1^ In developed nations, urothelial carcinoma (UC) comprises approximately 90% of subtypes of bladder cancer,^2^, ^3^, ^4^, ^5^ yet its incidence and prevalence in Japan are largely unknown.

Urothelial carcinoma is aggressive, and approximately 20% of patients presenting with UC and up to 50% of patients with UC undergoing radical surgery will develop metastases.^6^ The prognosis for metastatic UC (mUC) is poor, with overall survival of less than about 13 months and poor quality of life.^7^, ^8^, ^9^, ^10^

In the past decade, novel treatments, including programmed cell death protein 1/ligand 1 (PD‐1/L1) inhibitors and various antibody drug conjugates (ADCs), have been introduced.^11^, ^12^ Treatment guidelines in many countries recommend chemotherapy, including platinum‐based chemotherapy, as first‐line treatment for mUC,^13^, ^14^ PD‐1/L1 inhibitors as secondary or maintenance therapy,^15^ and ADCs and fibroblast growth factor receptor inhibitors as tertiary therapy.^11^, ^16^

In Japan, the PD‐1 inhibitor pembrolizumab was approved in December 2017 for second‐line treatment of mUC that has progressed following chemotherapy and cannot be surgically resected.^17^, ^18^ In 2021, the checkpoint inhibitor avelumab was approved as maintenance therapy, and the ADC enfortumab vedotin was approved for third‐line therapy. Although new treatments are now available, large‐scale studies examining epidemiology and shifts in treatment patterns after approval of immunotherapy for locally advanced or mUC (la/mUC) in Japan are scarce.

The primary objective of this study was to evaluate the epidemiologic trends (incidence and prevalence) in newly diagnosed patients (2015 and 2019) with la/mUC in Japan using real‐world data from a nationwide‐linked electronic medical record Diagnostic Procedure Combination claims dataset. Treatment patterns were evaluated as a secondary objective in newly diagnosed patients. Results were further compared with the time period before (2015–2017) and after (2018–2019) the approval of pembrolizumab for mUC in Japan.

## METHODS

### Data source and study population

This was a retrospective cohort study of data from the Health, Clinic, and Education Information Evaluation Institute and Real World Data Company database maintained by the Health, Clinic, and Education Information Evaluation Institute (Kyoto, Japan). The database comprises inpatient and outpatient data, including demographics, diagnoses, prescriptions, procedures, and laboratory results from approximately 20 million patients from 185 institutions across Japan as of December 2019. Patient‐level electronic medical record and Diagnostic Procedure Combination claims data (i.e., a health care payment system used in Japan and health care claims datasets) were linked with a unique identifier and anonymized at each medical institution.

**FIGURE 1:**
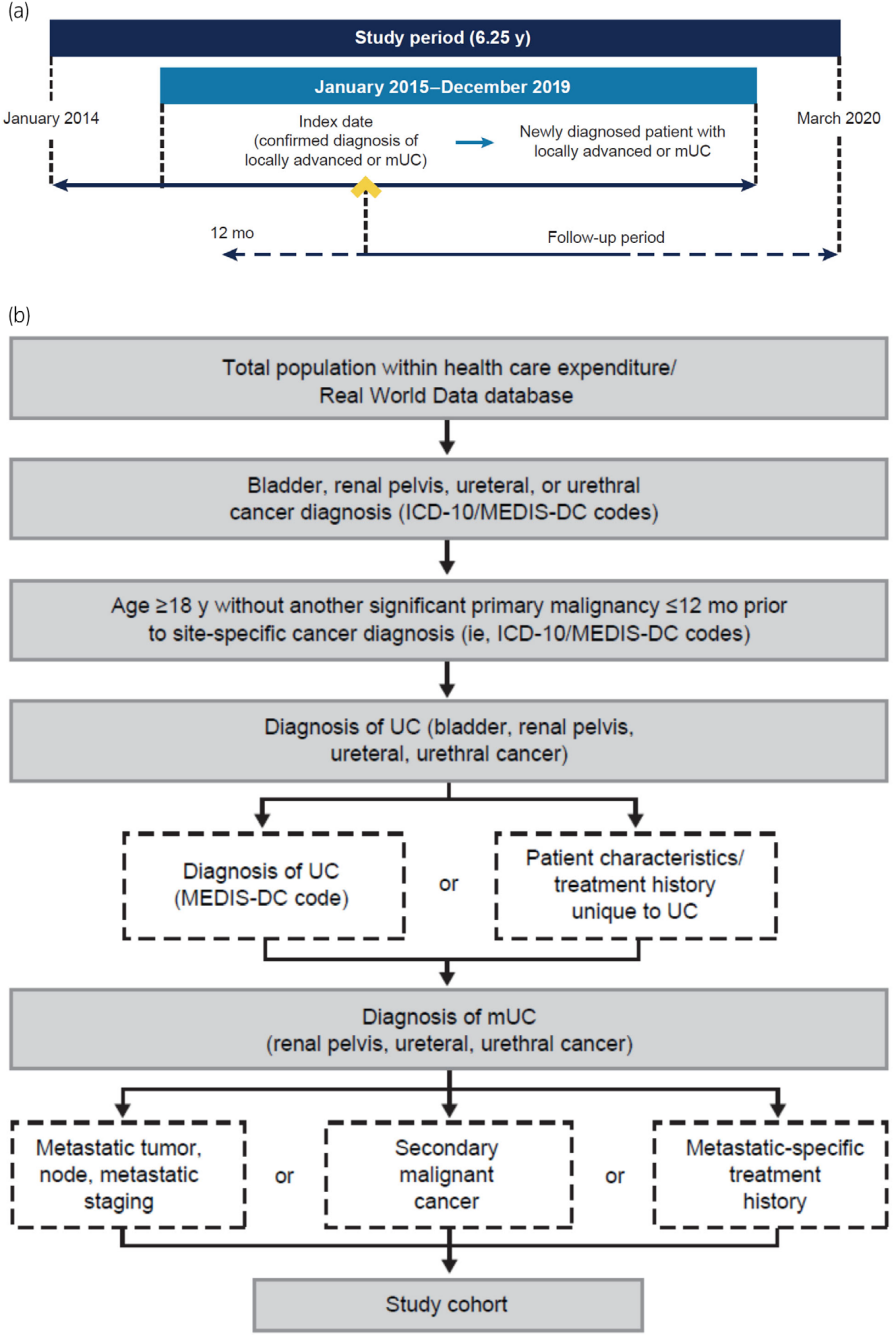
(a) Study design and (b) study population algorithm for patient volunteers with newly diagnosed locally advanced or mUC. (b) Multiple criteria are employed for steps 4 and 5 of deriving the patient cohort (e.g., to avoid biasing the population treated for mUC). ICD‐10, *International Classification of Diseases, Tenth Revision*; MEDIS‐DC, Medical Information System Development Center; mUC, metastatic urothelial carcinoma; UC, urothelial carcinoma.

Patients in the database with a diagnosis of bladder, renal pelvic, ureteric, or urethral cancer between January 2010 and December 2019 were screened. The study period was January 2014 to March 2020. Adults with the first record of la/mUC between 2015 and 2019 were included in this analysis (Figure 1a). The tumor cell type (UC) was defined through a staged algorithm that included UC‐specific Medical Information System Development Center codes, test results, and treatment history. Metastatic status was identified using an operational definition that included tumor, node, and metastasis staging, the presence of a secondary malignancy using *International Classification of Diseases, Tenth Revision*, codes, or other clinical coding and treatment regimens for la/mUC (Figure 1b). Patients who were part of a clinical trial were not excluded from analyses. Additional eligibility criteria can be found in the Data S1.

The index date (or incident event) was the date of the first record in the index period (January 2015–December 2019, inclusive) and met the definition for the diagnosis of la/mUC. All patients were observed from the index date until the date of their last database entry or the end of the study period (i.e., observation period), whichever occurred earlier. To analyze treatment patterns, incident patients were observed over a variable follow‐up period from the diagnosis of la/mUC until the date of their last database entry, death, or the end of the study period, whichever occurred first.

### Study outcomes

The primary endpoints were newly diagnosed cases (or rates) and prevalence (point and period) of la/mUC in Japan over time and the baseline patient demographics and clinical characteristics of these cohorts. Secondary endpoints were the first‐ and second‐line treatment patterns of patients newly diagnosed with la/mUC in Japan. These endpoints were chosen as they allow to estimate the number of cases that are eligible for secondary or tertiary therapies.

To assess changes in the epidemiology of la/mUC over time, the annual incidence proportion (number of new cases per calendar year as a proportion of the total population at risk in the database at the start of the calendar year) and annual incidence rate (number of new cases per calendar year divided by the sum of the total time the population within the database is at risk of disease in the calendar year) were calculated. The incidence risk and rate for the index period were also calculated. The point prevalence (number of patients with a confirmed diagnosis of la/mUC of the total population in the database) was also assessed on December 31 of each study year in addition to the period prevalence for each calendar year and for the index period. The period prevalence was the existing cases present at the start of each year and the new cases that arose during the year as a proportion of the total population in the database at any point during the calendar year. The incidence and prevalence were adjusted for age, sex, and Japanese region (Hokkaido, Tohoku, Kanto, Kinki, Chubu, Chugoku, Shikoku, Kyushu, and Okinawa) and were calculated and standardized to the national population level using 2015 Japan population census numbers. The crude incidence rate was reported per 100 000 person years; incidence risk and prevalence (point and period) were reported per 100 000. These were adjusted using the following formula,

$$\begin{array}{l} \text{Adjusted incidence or prevalence}\\ \;=\text{crude rate}\times \text{distribution, } \end{array}$$

where distribution was calculated as the population by stratification level (census) divided by total population (census).

A newly diagnosed (i.e., index) case was ascertained by confirming that the patient did not meet criteria for la/mUC in the dataset of the specific calendar year before their incident event. Patients remained classified as “prevalent” cases until they no longer met the criteria or until the end of the study.

Patient demographics and clinical characteristics were obtained for the newly diagnosed la/mUC during the 12 months prior to the index date. Treatment patterns during follow‐up were evaluated, starting from the date of la/mUC diagnosis until the end of the patient's observation period (date of their last database entry or end of study period). Variable follow‐up durations were used to assess treatments and calculate incidence and prevalence at different time points. For the analysis of treatment patterns, patients were stratified based on whether they received treatment for la/mUC before (January 2015–December 2017) or after approval (January 2018–December 2019) of pembrolizumab in Japan.

### Statistical analysis

Data were descriptively summarized. All analyses were stratified by age group, sex, and geographic region. Sensitivity analysis was conducted based on different methods of identifying metastatic status, as previously described. The Diagnostic Procedure Combination codes, laboratory tests based on Japanese Laboratory Test Code coding, Medical Information System Development Center codes, *International Classification of Diseases, Tenth Revision*, codes, and Anatomical Therapeutic Chemical (European Pharmaceutical Market Research Association; World Health Organization) codes were all used in this study. All data manipulation, variable derivations, and data analyses were conducted in Stata version 15 or later (StataCorp LLC, TX).

### Ethical statement

Institutional ethics approval and informed consent were not required because deidentified data were used. The Medical Affairs Japan Protocol Review Committee reviewed and approved the study protocol prior to study initiation.

## RESULTS

### Demographics and clinical characteristics

**FIGURE 2:**
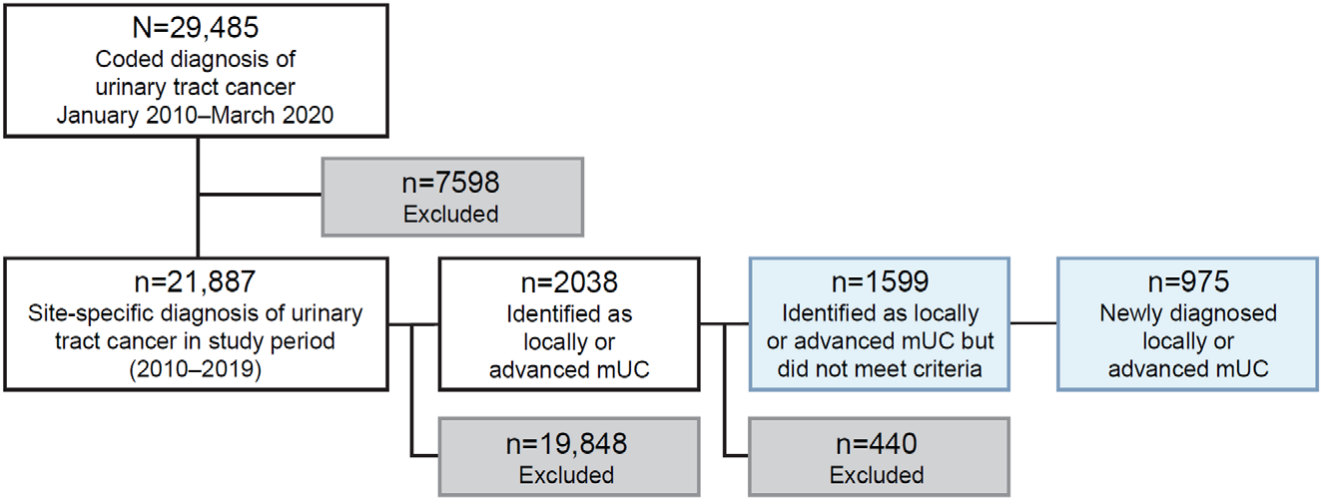
Composition of study patients with newly diagnosed locally advanced or mUC. mUC, metastatic urothelial carcinoma.

Of 29 485 patients identified with a diagnosis of bladder, renal pelvic, ureteric, or urethral cancer between January 2010 and March 2020, 975 met the definition of newly diagnosed la/mUC and were included (Figure 2). Most patients were men and the incidence of la/mUC increased with age (Table 1). A total of 71.6% of patients were aged 70 years or older; 62.8% of men in the study had a past or current history of smoking, whereas 58.3% of women had never smoked. Most patients were registered at hospitals in the Kinki region (37.5%), followed by the Tohoku (20.3%), Chubu (13.9%), and Chugoku (12.0%) regions.

**TABLE 1:** Patient demographics and clinical characteristics.

Parameter	Overall	Before pembrolizumab approval (2015–2017)	After pembrolizumab approval (2018–2019)
	*N* = 975	*n* = 307	*n* = 668
Mean age (SD), year	73.7 (9.0)	73.1 (9.4)	74.1 (8.8)
Age, year			
19–39	3 (0.3)	2 (0.7)	1 (0.1)
40–49	13 (1.3)	7 (2.3)	6 (0.9)
50–59	44 (4.5)	11 (3.6)	33 (4.9)
60–69	217 (22.3)	84 (27.4)	133 (19.9)
70–79	428 (43.9)	115 (37.5)	313 (46.9)
≥80	270 (27.7)	88 (28.7)	182 (27.2)
Sex			
Male	745 (76.4)	238 (77.5)	507 (75.9)
Female	230 (23.6)	69 (22.5)	161 (24.1)
Hospital region			
Chubu	136 (13.9)	48 (15.6)	88 (13.3)
Chugoku	117 (12.0)	9 (2.9)	108 (16.2)
Hokkaido	100 (10.3)	31 (10.1)	69 (10.3)
Kanto	17 (1.7)	8 (2.6)	9 (1.3)
Kinki	366 (37.5)	120 (39.1)	246 (36.8)
Kyushu or Okinawa	31 (3.2)	11 (3.6)	20 (3.0)
Shikoku	10 (1.0)	7 (2.3)	3 (0.4)
Tohoku	198 (20.3)	73 (23.8)	125 (18.7)
Smoking status at index			
Never smoked	281 (28.8)	93 (30.3)	188 (28.1)
History of smoking/Currently smoking	520 (53.3)	164 (53.4)	356 (53.3)
Unknown	174 (17.8)	50 (16.3)	124 (18.6)
Indexed tumor site			
Bladder	687 (70.5)	201 (65.5)	486 (72.8)
Ureter	154 (15.8)	52 (16.9)	102 (15.3)
Renal pelvis	123 (12.6)	48 (15.6)	75 (11.2)
Urethra	11 (1.1)	6 (2.0)	5 (0.7)
Metastases			
0/Unknown	318 (32.6)	75 (24.4)	243 (36.4)
1	348 (35.7)	130 (42.3)	218 (32.6)
≥2	309 (31.7)	102 (33.2)	207 (31.0)
Site of metastases			
Lymph node	283 (29.0)	100 (32.6)	183 (27.4)
Bone/Bone marrow	192 (19.7)	59 (19.2)	133 (19.9)
Digestive organ	232 (23.8)	89 (29.0)	143 (21.4)
Multiple sites	289 (29.6)	96 (31.3)	193 (28.9)
Respiratory organ	232 (23.8)	90 (29.3)	142 (21.3)
Skin	9 (0.9)	4 (1.3)	5 (0.7)
Adrenal gland	6 (0.6)	0 (0.0)	6 (0.9)
Bladder	20 (2.1)	8 (2.6)	12 (1.8)
Kidney	20 (2.1)	6 (1.9)	14 (2.1)
Nervous system	38 (3.9)	9 (2.9)	29 (4.3)
Other specified site	56 (5.7)	9 (2.9)	29 (4.3)
Ovary	1 (0.1)	0 (0.0)	1 (0.1)
Stage, kidney disease, *n*	857	266	591
1	12 (1.4)	6 (2.3)	6 (1.0)
2	291 (34.0)	93 (35.0)	198 (33.5)
3a	260 (30.3)	89 (33.5)	171 (28.9)
3b	227 (26.5)	61 (22.9)	166 (28.1)
4	67 (7.8)	17 (6.4)	50 (8.5)
Baseline Charlson Comorbidity Index, mean (SD)	6.9 (2.8)	7.4 (2.7)	6.7 (2.9)
Comorbidity			
Dementia	52 (5.3)	20 (6.5)	32 (4.8)
Cerebrovascular disease	230 (23.6)	77 (25.1)	153 (22.9)
Chronic kidney disease	93 (9.5)	30 (9.8)	63 (9.4)
Chronic obstructive pulmonary disease	238 (24.4)	76 (24.8)	162 (24.3)
Heart failure	267 (27.4)	79 (25.7)	188 (28.1)
Diabetes mellitus	434 (44.5)	115 (37.5)	319 (47.8)
Hearing loss	62 (6.4)	25 (8.1)	37 (5.5)
Hypertension	522 (53.5)	149 (48.5)	373 (55.8)
Irritable bowel disease/Crohn disease/Ulcerative colitis	45 (4.6)	8 (2.6)	37 (5.5)
Myocardial infarction	76 (7.8)	21 (6.8)	55 (8.2)
Neuropathy	162 (16.6)	55 (17.9)	107 (16.0)
Renal disease	59 (6.1)	18 (5.9)	41 (6.1)
Rheumatoid arthritis	29 (3.0)	13 (4.2)	16 (2.4)
Spinal cord injury	3 (0.3)	0 (0.0)	3 (0.4)
Stroke/Transient ischemic attack	192 (19.7)	64 (20.8)	128 (19.2)

*Note*: Values are *n* (%) unless otherwise indicated.

The majority of la/mUC tumors were lower tract malignancies (71.6%); of these, 98.4% were bladder carcinoma. Upper tract malignancies were evenly split between renal pelvic and ureteric tumors. Men had a greater proportion of bladder tumors than women (74.8% vs. 60.9%), whereas women had a higher proportion of upper tract tumors (39.1% vs. 25.1%). Patients with newly diagnosed la/mUC had a high number of comorbidities, and 77.3% had a Charlson Comorbidity Index score above 5.

Overall, 307 (31.5%) patients were newly diagnosed with la/mUC between 2015 and 2016, and 668 (68.5%) were diagnosed between 2017 and 2019. Demographics and disease characteristics were generally well balanced between the 2 treatment periods; however, a greater proportion of patients diagnosed between 2017 and 2018 were aged 70 years or older vs those diagnosed before approval of pembrolizumab (74.1% vs 66.2%, respectively; Table 1).

### Incidence and prevalence

**FIGURE 3:**
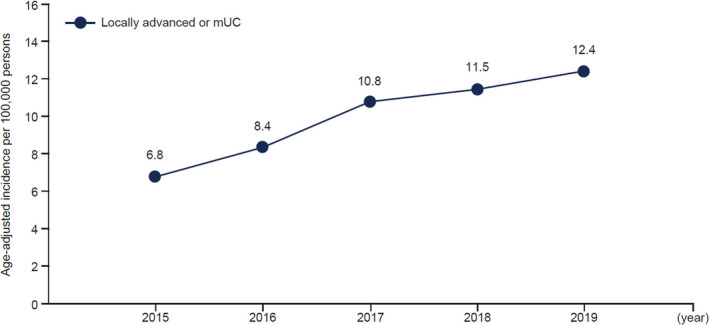
Age‐adjusted incidence over time of newly diagnosed locally advanced or mUC 2015–2019. mUC, metastatic urothelial carcinoma.

The annual newly diagnosed cases of la/mUC rose from 8.9 per 100 000 in 2015 to 18.9 per 100 000 people in 2019. Age‐adjusted incidence rates increased from 6.8 to 12.4 per 100 000 (Figure 3). For bladder cancer, between 2015 and 2019, age‐adjusted annual new cases increased from 4.2 to 9.3 per 100 000 persons; other urinary tract cases rose from 2.6 to 3.2 per 100 000.

**FIGURE 4:**
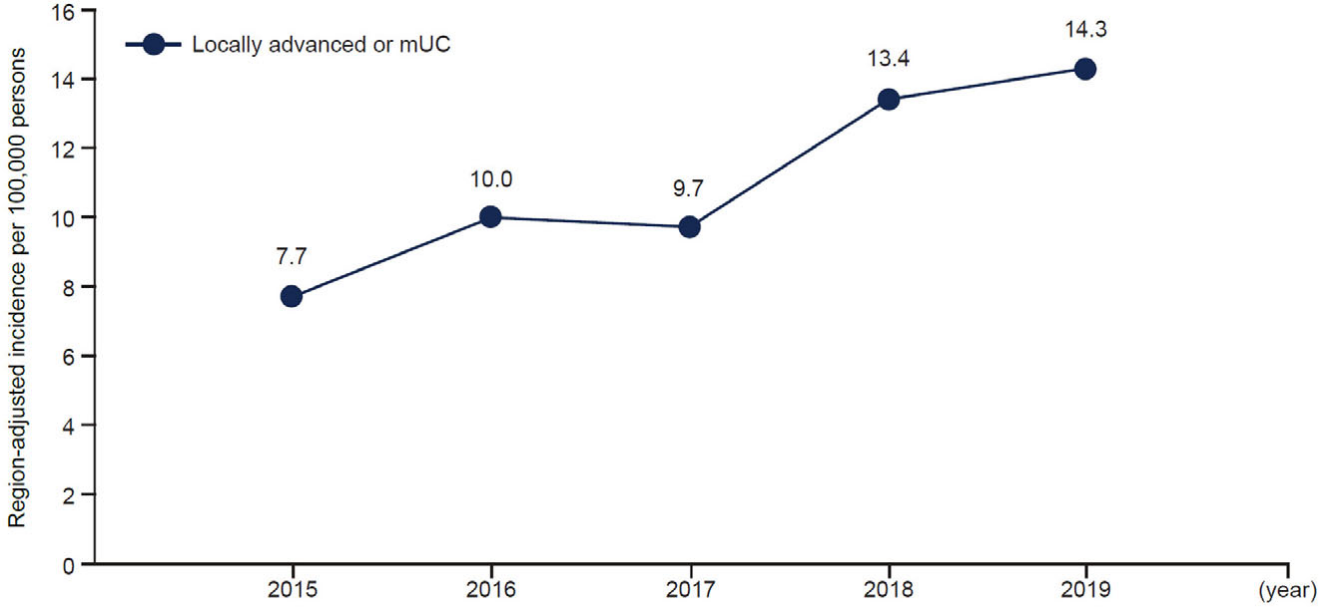
Region‐adjusted incidence over time of newly diagnosed locally advanced or mUC 2015–2019. mUC, metastatic urothelial carcinoma.

The 5‐year crude rate between 2015 and 2019 was 63.5 per 100 000 people, whereas age‐adjusted incidence during the same period was 47.8 per 100 000. Generally, the region‐adjusted incidence between 2015 and 2019 (50.5 per 100 000) was slightly higher than the age‐adjusted cases (Figure 4).

Age‐adjusted prevalence of all cases of la/mUC increased from 2015 to 2019, following a similar trend as incidence (Figure S1). The 5‐year crude prevalence between 2015 and 2019 was 38.9 per 100 000 people, whereas age‐adjusted prevalence was 33.2 per 100 000. The 5‐year region‐adjusted prevalence between 2015 to 2019 was 32.1 per 100 000 (Figure S2).

For bladder cancer, the age‐adjusted period prevalence rose from 8.8 per 100 000 in 2015 to 18.8 per 100 000 people in 2019; for other urinary tract cases, prevalence increased from 4.2 to 6.4 per 100 000.

### Treatment patterns

Of 975 patients in the UC cohort, 731 (75.0%) received systemic anticancer therapy. Of these 731 patients, 100% received 1 line and 50.2% received two lines of therapy (Table S1). The mean time from end of first line to start of second line was 3.61 months (95% confidence interval, 3.02–4.19). The proportion of newly diagnosed patients who received first‐line treatment increased from 69.4% (*n*/*N* = 213/307) to 77.5% (*n*/*N* = 518/668) before and after pembrolizumab approval periods, respectively.

The most common first‐line treatments in the 731 patients who received anticancer therapy were gemcitabine plus cisplatin or gemcitabine plus carboplatin; 78.3% of patients received either combination in the first‐line setting, a trend common across those initiating UC treatment before and after pembrolizumab approval for UC in Japan (Table 2). After pembrolizumab approval in 2017, an increase was observed in the proportion of patients who received first‐line treatment for newly diagnosed la/mUC (Table S1).

**TABLE 2:** First‐line treatment patterns in patients With newly diagnosed locally advanced or metastatic urothelial carcinoma.

Type of therapy^a^	Overall (2015–2019)	Before pembrolizumab approval (2015–2017)	After pembrolizumab approval (2018–2019)
	*N* = 731	(*n* = 213)	(*n* = 518)
Gemcitabine + platinum^b^	572 (78.3)	167 (78.4)	405 (78.2)
Taxane combination^c^	34 (4.7)	9 (4.2)	25 (4.8)
Other combination^d^	41 (5.6)	19 (8.9)	22 (4.2)
Pembrolizumab	16 (2.2)	0 (0)	16 (3.1)
Other monotherapy^e^	35 (4.8)	11 (5.2)	24 (4.6)
Other combination^f^	33 (4.5)	7 (3.3)	26 (5.0)

^a^ Defined in order of anticancer pharmacologic treatments after diagnosis of locally advanced or metastatic urothelial carcinoma.

^b^ Gemcitabine + cisplatin and gemcitabine + carboplatin.

^c^ Gemcitabine + platinum + docetaxel, platinum + paclitaxel, paclitaxel + others, and platinum + docetaxel.

^d^ Methotrexate, vinblastine sulfate, adriamycin, and cisplatin and platinum + others.

^e^ Platinum‐based therapy and others.

^f^ Including palliative care, lost to follow‐up (e.g., transfer to other hospitals).

Among 367 patients who received second‐line treatment, pembrolizumab was administered in 14.7% of patients; 22.3% of patients received gemcitabine plus cisplatin or gemcitabine plus carboplatin as second‐line treatment (Table 3). Baseline characteristics for patients receiving second‐line therapy were consistent with those receiving first‐line treatment and were also similar between before and after pembrolizumab approval (Table S2).

**TABLE 3:** Second‐line treatment patterns in patients with newly diagnosed locally advanced or metastatic urothelial carcinoma.

Type of therapy^a^	Overall (2015–2019)	Before pembrolizumab approval (2015–2017)	After pembrolizumab approval (2018–2019)
	*N* = 367	*n* = 121	*n* = 246
Gemcitabine + platinum^b^ ^,^ ^c^	82 (22.3)	31 (25.6)	51 (20.7)
Taxane combinations^d^	7 (1.9)	4 (3.3)	3 (1.2)
Other combinations^e^	60 (16.4)	24 (19.8)	36 (14.6)
Pembrolizumab	54 (14.7)	0 (0)	54 (22.0)
Other monotherapy^f^	111 (30.3)	41 (33.9)	70 (28.5)
Others^g^	53 (14.4)	21 (17.4)	32 (13.0)

^a^ Defined in order of anticancer pharmacologic treatments after diagnosis of locally advanced or metastatic urothelial carcinoma.

^b^ Includes triplets with platinum agents.

^c^ Gemcitabine + cisplatin and gemcitabine + carboplatin.

^d^ Gemcitabine + platinum + docetaxel, platinum + paclitaxel, paclitaxel + others, and platinum + docetaxel.

^e^ Methotrexate, vinblastine sulfate, adriamycin, and cisplatin, platinum + others, and paclitaxel + others.

^f^ Platinum, doxorubicin hydrochloride, and others.

^g^ All other antineoplastic agents Anatomical Therapeutic Chemical code L01, not otherwise mentioned.

## DISCUSSION

By analyzing real‐world data, this study provides insights into the epidemiology of newly diagnosed la/mUC in Japan and into the shift in treatment patterns observed in recent years due to access to novel therapies such as PD‐1/L1 inhibitors. The results suggest increases in the incidence and prevalence of la/mUC in Japan over time. The increasing age at UC diagnosis and the predominance of men among patients with UC in this study are in line with worldwide trends and previous research using data from the National Cancer Registry of Japan.^1^, ^19^, ^20^, ^21^

However, when adjusted for age, the increasing trend in incidence found in this study was less pronounced and less aligned with previously published data.^1^, ^6^ Based on the latter, age‐standardized incidence rates for newly diagnosed locally advanced or mUC are 2.88 and 0.58, for women and men, respectively, per 100 000 persons.^1^, ^6^ However, the present study demonstrated a rate of 6.8 cases per 100 000 persons in 2015, increasing to 12.4 cases per 100 000 persons in 2019, which is up to approximately 4 and 20 times the previously published rates for men and women, respectively. An incidence rate of 2.8 per 100 000 persons for la/mUC in Japan was reported previously, in the period before the approval of pembrolizumab, corroborating the trend observed in this study.^22^

Treatment patterns for la/mUC observed in this study are aligned with treatment guidelines and with published studies on Japanese and other international UC patient populations.^17^, ^23^, ^24^ A substantial proportion of patients (≈25%) in this study did not record any anticancer treatment, which is in line with Japanese research reporting that 9.3%–57.4% of patients with la/mUC do not receive systemic therapy.^25^, ^26^, ^27^ Similar rates (55%) have been reported for US patients with mUC.^28^ Platinum‐based regimens were most frequently used in patients eligible to receive them. Among patients receiving first‐line treatment, most (78.3%) received gemcitabine and platinum‐based chemotherapy. This is somewhat consistent with the 50.5%–87.9% rates reported for first‐line platinum‐based regimens in two retrospective Japanese studies of patients with mUC.^29^, ^30^

Few studies have investigated use of immunotherapy in the real‐world setting in Japan.^31^ The present study demonstrates a shift in treatment pattern after pembrolizumab was approved as second‐line treatment for newly diagnosed la/mUC in Japan. Only 2.2% of patients received first‐line pembrolizumab during the study period, possibly due to trial enrollment, compassionate extension of trials, or private use. Consistent with data reported for Japan previously,^32^ we observed an increase in the proportion of patients receiving second‐line systemic treatment in the period after versus before pembrolizumab approval.

The study has several limitations. Biases cannot be ruled out because the study used secondary data from real‐world clinical settings that were recorded for purposes other than observational research. However, the cohort design and use of clear case definitions and a logical algorithm could have mitigated some of the limitations of using secondary data. Patients who moved to other hospitals could not be tracked in this dataset; thus, epidemiologic estimates may not be precise. Moreover, no standardized definitions (i.e., validated code sets) for la/mUC diagnosis are available; therefore, study‐specific code lists and algorithms were developed to identify patients with newly diagnosed la/mUC in this study. This could have led to inconsistent results between studies using the same data and could also have resulted in underestimation of the number of events. In addition, use of diagnostic codes for case identification could potentially have led to underestimation of the epidemiology of UC because some clinicians do not use diagnostic codes in practice or do not update them as the patient's disease progresses. Moreover, prescription data may not have accurately reflected medication use. To simplify the analysis, similar therapies to those used for newly diagnosed la/mUC were grouped into similar treatment classes. Furthermore, due to the descriptive nature of the analyses used in the study, there is a risk of potential confounding. Patients in this database were collected from selected prefectures in Japan, resulting in potential lack of generalizability of the study results. Of note, patients could also have exited the data source for reasons other than death or end of the study period. Tracking patients between different hospitals was not possible; therefore, a patient who received care at two different centers may have been counted twice if both centers were in the database.

By using real‐world data of approximately 20 million patients from 185 institutions, our results show increases in the incidence and prevalence of la/mUC in Japan. Although the demographics and clinical characteristics were similar before and after pembrolizumab approval in Japan, first‐line treatment rates increased after pembrolizumab approval. High attrition rates across lines of therapy were observed, suggesting an unmet treatment need in this patient population.

## AUTHOR CONTRIBUTIONS

**Keiko Asakawa**: Contribution, study design, data interpretation, manuscript preparation, writing—review & editing. **Miina Waratani**: Contribution, study design, collection and assembly of data, data analysis, data interpretation, manuscript preparation, writing—review & editing. **Olivia Massey**: Data analysis, data interpretation, manuscript preparation, writing—review & editing. **Tim Holbrook**: Contribution, study design, manuscript preparation, writing—review & editing. **Makoto Kondo**: Contribution, study design, data interpretation, manuscript preparation, writing—review & editing. **Atsushi Saito**: Contribution, study design, data interpretation, manuscript preparation, writing—review & editing. **Hiroyuki Nishiyama**: Contribution, study design, data interpretation, manuscript preparation, writing—review & editing. All authors participated in the critical review and revision of this manuscript and provided approval of the manuscript for submission.

## CONFLICT OF INTEREST STATEMENT

Keiko Asakawa is an employee of Astellas Pharma, Inc., and declares no other conflict of interest. Miina Waratani is an employee of Astellas Pharma, Inc., and declares no other conflict of interest. Olivia Massey reports that employer, Adelphi Real World, received funding from Astellas Pharma, Inc., to design and conduct the study. Tim Holbrook has nothing to report. Makoto Kondo has nothing to report. Atsushi Saito is an employee of Astellas Pharma, Inc., and declares no other conflict of interest. Hiroyuki Nishiyama reports grants from Astellas Pharma, Inc., Ono Pharmaceutical, Takeda Pharmaceutical, and Bayer; and reports payment or honoraria for speakers bureaus from MSD, Chugai Pharmaceutical, and Olympus.

## REGISTRY AND THE REGISTRATION NO. OF THE STUDY/TRIAL

N/A.

## ANIMAL STUDIES

N/A.

## FUNDING INFORMATION

This study is sponsored by Astellas Pharma, Inc., and Seagen, which was acquired by Pfizer in Dec. 2023.

## ETHICS STATEMENT

Institutional ethics approval and informed consent were not required because deidentified data were used. The Medical Affairs Japan Protocol Review Committee reviewed and approved the study protocol prior to study initiation.

## Supporting information


Data S1.
Table S1.
Table S2.
Figure S1.
Figure S2.

## Data Availability

Researchers may request access to anonymized participant level data, trial level data and protocols from Astellas sponsored clinical trials at www.clinicalstudydatarequest.com. For the Astellas criteria on data sharing see: https://clinicalstudydatarequest.com/Study‐Sponsors/Study‐Sponsors‐Astellas.aspx.
