# Supplementary material for: Real‐world epidemiology and treatment patterns of patients with locally advanced or metastatic urothelial carcinoma: Retrospective analysis of Diagnosis Procedure Combination claims data in Japan
Source: Int J Urol. 2024 Mar 12;31(7):730–8. doi: 10.1111/iju.15450 (PMC11524106; doi:10.1111/iju.15450)

**SUPPLEMENTARY MATERIAL**

**Inclusion Criteria**

- Diagnosis of bladder cancer (C67: malignant neoplasm of bladder), renal pelvis cancer (C65: malignant neoplasm of renal pelvis), ureteral cancer (C66: malignant neoplasm of ureter), urethral cancer (C68[.0]: malignant neoplasm of other and unspecified urinary organs [urethra]) according to the appropriate *International Classification of Diseases, Tenth Revision* (ICD-10), codes or equivalent Medical Information System Development Center (MEDIS-DC) codes
- Aged 18 years of age or older on or prior to a date of diagnosis of bladder, renal pelvis, ureteral, or urethral cancer
- Urothelial carcinoma (UC) histopathologic subtype with either
  - MEDIS-DC UC diagnosis code
  - Patient characteristics/Treatment history unique to UC
- Presence of metastatic disease identified through a combination of either:
  - Tumor, node, metastasis staging
  - Presence of secondary malignancies according to appropriate ICD-10/equivalent MEDIS-DC codes
  - Receiving treatment with any of the following:
    - Pembrolizumab
    - Paclitaxel
    - Docetaxel
    - Carboplatin
    - Cisplatin

**Exclusion Criteria**

- Primary diagnosis of other significant solid primary tumor malignancies in the 12 months prior to site-specific cancer diagnosis (ie, bladder, ureteral, urethral, renal pelvis cancer) date according to appropriate ICD-10/equivalent MEDIS-DC codes
- Confirmed nonurothelial urinary cancer according to appropriate MEDIS-DC codes (8840140: bladder sarcoma; 8845337: renal pelvic squamous cell carcinoma; 8845494: bladder squamous cell carcinoma), test results, and patient characteristics or treatment history
- Diagnosis of carcinoma of the urachus (ICD-10: C67.7) any time during the observation period
- Treatment with a tyrosine kinase inhibitor any time during the observation period
- Not treated with any other chemotherapy except for neoadjuvant/adjuvant treatment with cisplatin
  - Neoadjuvant therapy is cisplatin treatment before cystectomy
  - Adjuvant therapy is cisplatin treatment starting within 3 months from cystectomy

**Table S1.** Patient characteristics at baseline: First-line treatment

| **Characteristic** | **All periods (2015–2019)**  **(n=731)** | | | | | **Before pembrolizumab approval (2015–2017)**  **(n=213)** | | | | | **After pembrolizumab approval (2018–2019)**  **(n=518)** | | | | |
| --- | --- | --- | --- | --- | --- | --- | --- | --- | --- | --- | --- | --- | --- | --- | --- |
|  | **Overall** | **Gemcitabine combo^†^** | **Taxane combo** | **Other combo** | **Other mono** | **Overall** | **Gemcitabine combo^†^** | **Taxane combo** | **Other combo** | **Other mono** | **Overall** | **Gemcitabine combo^†^** | **Taxane combo** | **Other combo** | **Other mono** |
| **Age group, y** | | | | | | | | | | | | | | | |
| n | 731 | 229 | 34 | 451 | 17 | 213 | 69 | 9 | 128 | 7 | 518 | 160 | 25 | 323 | 10 |
| <70 | 384 (52.5) | 122 (53.3) | 16 (47.1) | 236 (52.3) | 10 (58.8) | 127 (59.6) | 42 (60.9) | 7 (77.8) | 74 (57.8) | 4 (57.1) | 257 (49.6) | 80 (50.0) | 9 (36.0) | 162 (50.2) | 6 (60.0) |
| 70–79 | 347 (47.5) | 107 (46.7) | 18 (52.9) | 215 (47.7) | 7 (41.2) | 86 (40.4) | 27 (39.1) | 2 (22.2) | 54 (42.2) | 3 (42.9) | 261 (50.4) | 80 (50.0) | 16 (64.0) | 161 (49.9) | 4 (40.0) |
| **Kidney disease stage** | | | | | | | | | | | | | | | |
| n | 647 | 208 | 30 | 393 | 16 | 189 | 59 | 9 | 115 | 6 | 458 | 149 | 21 | 278 | 10 |
| 1–2 | 229 (35.4) | 33 (15.9) | 14 (46.7) | 172 (43.8) | 10 (62.5) | 70 (37.1) | 11 (18.6) | 3 (33.3) | 53 (46.1) | 3 (50.0) | 159 (34.7) | 22 (14.8) | 11 (52.4) | 119 (42.8) | 7 (70.0) |
| 3a | 214 (33.1) | 62 (29.8) | 8 (26.7) | 141 (35.9) | 3 (18.8) | 68 (36.0) | 19 (32.2) | 2 (22.2) | 45 (39.1) | 2 (33.3) | 146 (31.9) | 43 (28.9) | 6 (28.6) | 96 (34.5) | 1 (10.0) |
| 3b/4 | 204 (31.5) | 113 (54.3) | 8 (26.7) | 80 (20.4) | 3 (18.8) | 51 (27.0) | 29 (49.2) | 4 (44.4) | 17 (14.8) | 1 (16.7) | 153 (33.4) | 84 (56.4) | 4 (19.1) | 63 (22.7) | 2 (20.0) |
| **ECOG PS** | | | | | | | | | | | | | | | |
| n | 594 | 177 | 18 | 385 | 14 | 191 | 59 | 7 | 120 | 5 | 403 | 118 | 11 | 265 | 9 |
| Severe dependency | 9 (1.52) | 2 (1.1) | 1 (5.6) | 5 (1.3) | 1 (7.1) | 3 (1.6) | 2 (3.4) | 0 (0) | 0 (0) | 1 (20.0) | 6 (1.5) | 0 (0) | 1 (9.1) | 5 (1.9) | 0 (0) |
| Moderate dependency | 3 (0.51) | 0 (0) | 0 (0) | 2 (0.5) | 1 (7.1) | 1 (0.5) | 0 (0) | 0 (0) | 0 (0) | 1 (20.0) | 2 (0.5) | 0 (0) | 0 (0) | 2 (0.8) | 0 (0) |
| Mild/no dependency | 582 (98.0) | 175 (98.9) | 17 (94.4) | 378 (98.2) | 12 (85.7) | 187 (97.9) | 57 (96.6) | 7 (100) | 120 (100) | 3 (60.0) | 395 (98.0) | 118 (100) | 10 (90.9) | 258 (97.4) | 9 (100) |

*Note*: All values are n (%) unless otherwise indicated. ^†^Gemcitabine + cisplatin and gemcitabine + carboplatin.

**Table S2.** Patient characteristics at baseline: Second-line treatment

| **Characteristic** | **All periods (2015–2019)**  **(n=367)** | | | | | **Before pembrolizumab approval (2015–2017)**  **(n=121)** | | | | | **After pembrolizumab approval (2018–2019)**  **(n=246)** | | | | |
| --- | --- | --- | --- | --- | --- | --- | --- | --- | --- | --- | --- | --- | --- | --- | --- |
|  | **Overall** | **Gemcitabine combo^†^** | **Taxane combo** | **Other combo** | **Other mono** | **Overall** | **Gemcitabine combo^†^** | **Taxane combo** | **Other combo** | **Other mono** | **Overall** | **Gemcitabine combo^†^** | **Taxane combo** | **Other combo** | **Other mono** |
| **Age, y, n (%)** | | | | | | | | | | | | | | |  |
| n | 367 | 120 | 15 | 223 | 9 | 121 | 43 | 5 | 69 | 4 | 246 | 77 | 10 | 154 | 5 |
| <70 | 188 (51.2) | 69 (57.5) | 8 (53.3) | 107 (48.0) | 4 (44.4) | 69 (57.0) | 26 (60.5) | 4 (80.0) | 37 (53.6) | 2 (50.0) | 119 (48.4) | 43 (55.8) | 4 (40.0) | 70 (45.5) | 2 (40.0) |
| 70–79 | 179 (48.8) | 51 (42.5) | 7 (46.7) | 116 (52.0) | 5 (55.6) | 52 (43.0) | 17 (39.5) | 1 (20.0) | 32 (46.4) | 2 (50.0) | 127 (51.6) | 34 (44.2) | 6 (60.0) | 84 (54.6) | 3 (60.0) |
| **Stage of kidney disease**^§^**, n (%)** | | | | | | | | | | | | | | | |
| n | 321 | 107 | 14 | 192 | 8 | 102 | 34 | 5 | 60 | 3 | 219 | 73 | 9 | 132 | 5 |
| 1–2 | 128 (39.9) | 23 (21.5) | 8 (57.1) | 91 (47.4) | 6 (75.0) | 41 (40.2) | 9 (26.5) | 3 (60.0) | 28 (46.7) | 1 (33.3) | 87 (39.7) | 14 (19.2) | 5 (55.6) | 63 (47.7) | 5 (100) |
| 3a | 103 (32.1) | 33 (30.8) | 2 (14.3) | 67 (34.9) | 1 (12.5) | 39 (38.2) | 13 (38.2) | 1 (20.0) | 24 (40.0) | 1 (33.3) | 64 (29.2) | 20 (27.4) | 1 (11.1) | 43 (32.6) | 0 (0) |
| 3b or 4 | 90 (28.0) | 51 (47.7) | 4 (28.6) | 34 (17.7) | 1 (12.5) | 22 (21.6) | 12 (35.3) | 1 (20.0) | 8 (13.3) | 1 (33.3) | 68 (31.1) | 39 (53.4) | 3 (33.3) | 26 (19.7) | 0 (0) |
| **ECOG PS**^§^**, n (%)** | | | | | | | | | | | | | | | |
| n | 311 | 102 | 11 | 191 | 7 | 109 | 40 | 4 | 63 | 2 | 202 | 62 | 7 | 128 | 5 |
| Severe dependency | 2 (0.6) | 0 (0) | 0 (0) | 2 (1.1) | 0 (0) | 0 (0) | 0 (0) | 0 (0) | 0 (0) | 0 (0) | 2 (1.0) | 0 (0) | 0 (0) | 2 (1.6) | 0 (0) |
| Moderate dependency | 1 (0.3) | 0 (0) | 0 (0) | 0 (0) | 1 (14.3) | 1 (0.9) | 0 (0) | 0 (0) | 0 (0) | 1 (50.0) | 0 (0) | 0 (0) | 0 (0) | 0 (0) | 0 (0) |
| Mild dependency/ None | 308 (99.0) | 102 (100) | 11 (100) | 189 (99.0) | 6 (85.7) | 108 (99.1) | 40 (100) | 4 (100) | 63 (100) | 1 (50.0) | 200 (99.0) | 62 (100) | 7 (100) | 126 (98.4) | 5 (100) |

*Note*: All values are n (%) unless otherwise indicated. ^†^Gemcitabine + cisplatin and gemcitabine + carboplatin. ^§^Not collected at second-line treatment, so these values could have changed since baseline (results should be interpreted with caution).

**Figure S1.** Age-adjusted prevalence of newly diagnosed locally advanced or mUC 2015–2019. Abbreviation: mUC, metastatic urothelial carcinoma.


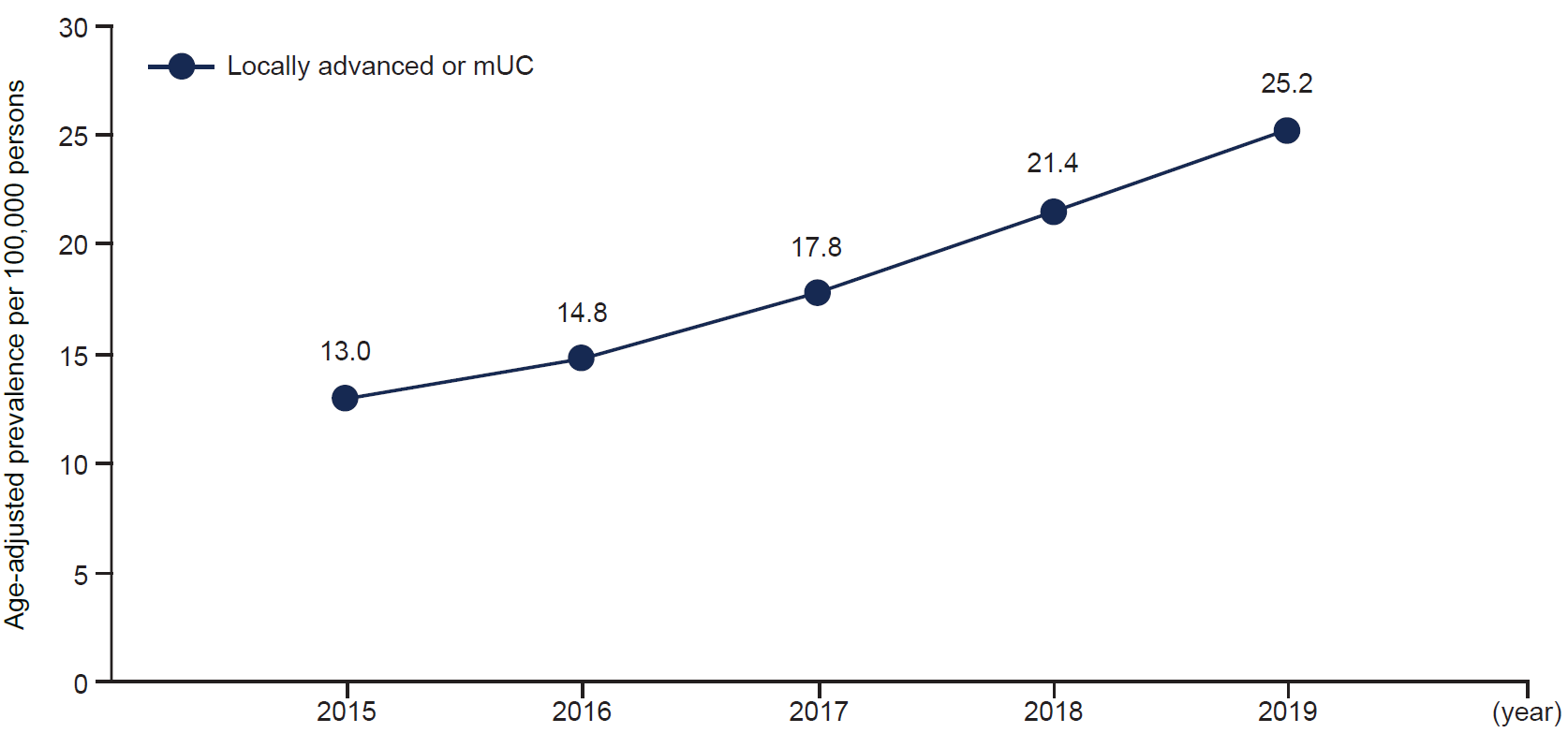


**Figure S2.** Region-adjusted prevalence of newly diagnosed locally advanced or mUC 2015–2019. Abbreviation: mUC, metastatic urothelial carcinoma.


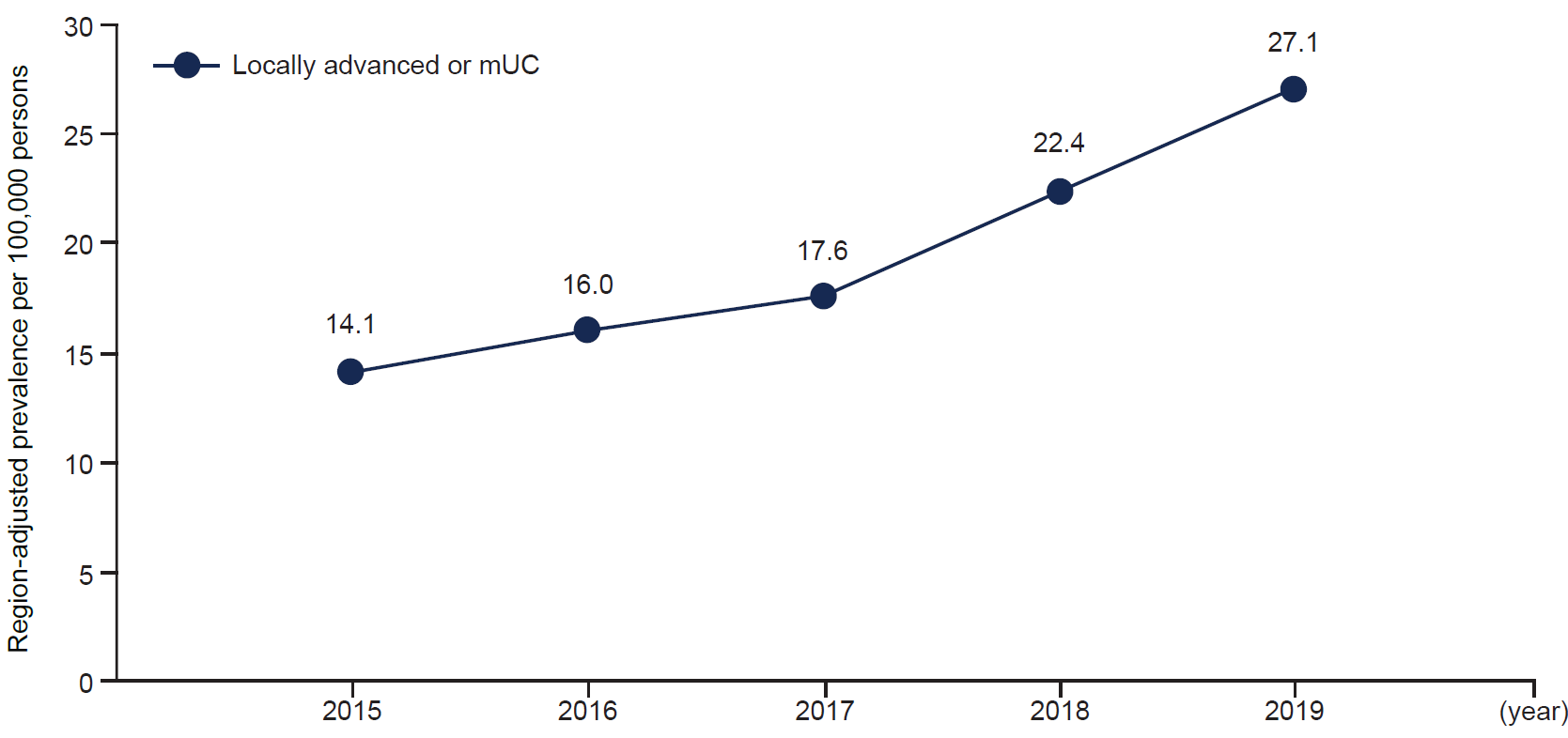

Supplement: Supplementary file 1 — Data S1. Table S1. Table S2. Figure S1. Figure S2. [file IJU-31-730-s001.docx]
